# Impact of HIV-1 Subtype and Antiretroviral Therapy on Protease and Reverse Transcriptase Genotype: Results of a Global Collaboration

**DOI:** 10.1371/journal.pmed.0020112

**Published:** 2005-04-26

**Authors:** Rami Kantor, David A Katzenstein, Brad Efron, Ana Patricia Carvalho, Brian Wynhoven, Patricia Cane, John Clarke, Sunee Sirivichayakul, Marcelo A Soares, Joke Snoeck, Candice Pillay, Hagit Rudich, Rosangela Rodrigues, Africa Holguin, Koya Ariyoshi, Maria Belen Bouzas, Pedro Cahn, Wataru Sugiura, Vincent Soriano, Luis F Brigido, Zehava Grossman, Lynn Morris, Anne-Mieke Vandamme, Amilcar Tanuri, Praphan Phanuphak, Jonathan N Weber, Deenan Pillay, P. Richard Harrigan, Ricardo Camacho, Jonathan M Schapiro, Robert W Shafer

**Affiliations:** **1**Division of Infectious Disease and Center for AIDS Research, Stanford UniversityStanford, CaliforniaUnited States of America; **2**Department of Statistics and Division of Biostatistics, Stanford UniversityStanford, CaliforniaUnited States of America; **3**Hospital Egas MonizLisbonPortugal; **4**BC Centre for Excellence in HIV/AIDS, VancouverBritish ColumbiaCanada; **5**Health Protection AgencyPorton DownUnited Kingdom; **6**Wright Fleming Institute, Imperial CollegeSt. Mary's Hospital, LondonUnited Kingdom; **7**Chulalongkorn UniversityBangkokThailand; **8**Universidade Federal do Rio de JaneiroBrazil; **9**Rega Institute for Medical ResearchLeuvenBelgium; **10**National Institute of Communicable DiseasesJohannesburgSouth Africa; **11**Central Virology, Public Health LaboratoriesMinistry of Health, Tel-HashomerIsrael; **12**Instituto Adolfo LutzSao PauloBrazil; **13**Hospital Carlos IIIMadridSpain; **14**National Institute of Infectious DiseasesTokyoJapan; **15**Fundación HuespedBuenos AiresArgentina; **16**University College London and Health Protection AgencyLondonUnited Kingdom; The Rockefeller UniversityUnited States of America

## Abstract

**Background:**

The genetic differences among HIV-1 subtypes may be critical to clinical management and drug resistance surveillance as antiretroviral treatment is expanded to regions of the world where diverse non-subtype-B viruses predominate.

**Methods and Findings:**

To assess the impact of HIV-1 subtype and antiretroviral treatment on the distribution of mutations in protease and reverse transcriptase, a binomial response model using subtype and treatment as explanatory variables was used to analyze a large compiled dataset of non-subtype-B HIV-1 sequences. Non-subtype-B sequences from 3,686 persons with well characterized antiretroviral treatment histories were analyzed in comparison to subtype B sequences from 4,769 persons. The non-subtype-B sequences included 461 with subtype A, 1,185 with C, 331 with D, 245 with F, 293 with G, 513 with CRF01_AE, and 618 with CRF02_AG. Each of the 55 known subtype B drug-resistance mutations occurred in at least one non-B isolate, and 44 (80%) of these mutations were significantly associated with antiretroviral treatment in at least one non-B subtype. Conversely, of 67 mutations found to be associated with antiretroviral therapy in at least one non-B subtype, 61 were also associated with antiretroviral therapy in subtype B isolates.

**Conclusion:**

Global surveillance and genotypic assessment of drug resistance should focus primarily on the known subtype B drug-resistance mutations.

## Abstract 2

Most research has focused on HIV-1 subtype B, although most infections worldwide are non-B. However, this paper suggests that the same drug resistance mutations occur across subtypes

## Introduction

The HIV-1 pandemic resulted from the cross-species transmission of a lentivirus, most likely of chimpanzee origin, that began spreading among humans during the first half of the previous century [1,2,3]. The progeny of this zoonotic infection—designated HIV-1 group M (main) viruses—make up the vast majority of HIV-1 infections. During their spread among humans, group M viruses have developed an extraordinary degree of genetic diversity, and most can be segregated into nine pure subtypes and several commonly circulating recombinant forms [4].

HIV-1 subtype B is the predominant subtype in North America, Western Europe, and Australia. The antiretroviral drugs used to treat HIV were developed using biophysical, biochemical, and in vitro studies of subtype B isolates, and most data on the genetic mechanisms of HIV-1 drug resistance are from subtype B viruses. However, HIV-1 subtype B viruses account for only approximately 12% of the global HIV pandemic [5], and as therapy is introduced into developing countries, the number of persons with non-B viruses initiating therapy will increase dramatically.

HIV-1 subtypes differ from one another by 10%–12% of their nucleotides and 5%–6% of their amino acids in protease and reverse transcriptase (RT) [6]. Intersubtype nucleotide differences influence the spectrum of amino acid substitutions resulting from point mutations, and intersubtype amino acid differences influence the biochemical and biophysical microenvironment within the protease and RT [7,8]. These differences among subtypes therefore could influence the spectrum of mutations that develop during selective drug pressure.

An increasing number of observational studies, in vitro and in vivo, suggest that the currently available protease and RT inhibitors are as active against non-B viruses as they are against subtype B viruses [9,10,11,12,13,14,15,16,17,18,19,20,21,22,23,24,25,26]. However, fewer data are available on the genetic mechanisms of drug resistance in non-B viruses, and some in vitro and in vivo observations suggest that the various subtypes may respond differently to certain antiretroviral drugs [27,28,29,30,31,32,33,34,35].

Identifying the relevant drug-resistance mutations among non-B subtypes will be important for monitoring the evolution and transmission of drug resistance, for determining initial treatment strategies for persons infected with non-B viruses, and for interpreting genetic resistance among patients who fail antiretroviral therapy.

In this study, we characterize protease and RT mutations in non-B HIV-1 subtypes from persons receiving antiretroviral therapy, and attempt to answer the following two questions. (i) Do the mutations that cause drug resistance in subtype B viruses also develop in non-B viruses exposed to antiretroviral drugs? (ii) Do novel mutations emerge in non-subtype-B viruses during antiretroviral drug failure that are not recognized in subtype B viruses?

## Methods

### HIV-1 Sequences and Antiretroviral Treatments

Sequences of HIV-1 protease (positions 1–99) and RT (positions 1–240) from persons whose antiretroviral treatment history was known were collected from the published literature and from 14 laboratories in 12 countries. Persons were considered untreated if they had never been exposed to antiretroviral drugs, and treated if they were receiving RT inhibitors (RTIs) and/or protease inhibitors (PIs) at the time the isolate was obtained. Sequences from treated persons were included for analysis only if they were obtained from persons whose entire treatment histories were known. If multiple isolates from the same person were sequenced, only the latest isolate was included for analysis. Only sequences determined using dideoxyterminator sequencing were included in the analysis. In all, 99% of sequences were determined using direct PCR (population-based sequencing), and 1% of sequences represented the consensus sequence of multiple clones.

Samples obtained from patients were submitted to clinical and research laboratories for resistance testing in the course of evaluation and care of HIV infection. Data analyzed included published and presented data obtained under protocols approved by national and local institutional review boards or ethical review panels in each country. Sequence, demographic, and treatment data, unlinked from all personal identifiers, were analyzed at Stanford University under a protocol approved by the Stanford University Panel on Human Subjects.

### Subtype Assignment

Similarity plotting and bootscanning using a window size of 400 nucleotides and a step size of 40 nucleotides were performed using reference sequences for each of the nine pure subtypes (A, B, C, D, F, G, H, J, and K) and two recombinant forms (CRF01_AE and CRF02_AG) [36]. Isolates that contained a combination of more than one subtype were excluded from analysis, except when subtypes A and G were detected in a pattern consistent with CRF02_AG. Because CRF01_AE *pol* sequences do not contain recombinant breakpoints, subtype assignment was based on the fact that *pol* CRF01_AE and pure A sequences are divergent. This approach had an accuracy of 96% when applied to the protease and RT genes of 137 well characterized subtype A, CRF01_AE, and CRF02_AG isolates with known subtypes based on *pol* and *gag* and/or *env,* with most errors resulting from the misclassification of subtype A protease sequences as CRF01_AE (data not shown).

Reference sequences used were U455 (subtype A), CM240 (CRF01_AE), IbNG (CRF02_AG), HXB2 (subtype B), C2220 (subtype C), NDK (subtype D), 93BR020 (subtype F), SE6165 (subtype G), 90CR056 (subtype H), SE9173c (subtype J), 97EQTB11C (subtype K), YBF30 (Group N), and ANT70C (Group O). A total of 223 protease and 307 RT sequences of indeterminate subtype were excluded from the analysis.

### Mutation Definitions

Each sequence was translated and compared to the consensus B protease and RT sequences in the Los Alamos HIV Sequence database (http://hiv-web.lanl.gov) using the HIVSeq program [37]. Mutations were defined as differences from the wild-type consensus B sequence. Known subtype B drug-resistance mutations were defined as follows: 18 nucleoside RTI (NRTI)–resistance positions at 41, 44, 62, 65, 67, 69, 70, 74, 75, 77, 115, 116, 118, 151, 184, 210, 215, and 219; 15 non-nucleoside RTI (NNRTI)–resistance positions at 98, 100, 101, 103, 106, 108, 179, 181, 188, 190, 225, 227, 230, 236, and 238; and 22 protease inhibitor (PI)–resistance positions at 10, 20, 24, 30, 32, 33, 36, 46, 47, 48, 50, 53, 54, 63, 71, 73, 77, 82, 84, 88, 90, and 93 [38,39]. Mutations also included differences from consensus B that were present as part of a nucleotide mixture.

Polymorphisms were defined as mutations that occurred in more than 1% of sequences from untreated persons. Subtype-specific polymorphisms were defined as mutations that were significantly more prevalent in each non-B subtype than in subtype B viruses from untreated persons. Subtype-specific treatment-related mutations were defined as mutations that were significantly more prevalent in HIV-1 isolates from treated persons than in isolates from untreated persons infected with the same subtype.

### Quality Control

Phylogenetic analysis to detect duplicate sequences identified 23 pairs of identical sequences and 1,039 pairs of sequences that differed from one another by no more than 1% of their nucleotides. To reduce the likelihood that isolates from different persons with similar mutations resulted from duplicate reporting, laboratory contamination, or HIV-1 transmission, only one sequence from each of these 1,062 sequence pairs was included in the analyses in this study. Although all extant HIV-1 isolates are epidemiologically linked through chains of transmission, protease or RT sequences that diverge by 1% or less appear to be more likely to result from direct transmission than those that diverge by more than 1% [40].

To distinguish mutations developing in multiple individuals from mutations that developed in a smaller number of founder viruses, we reconstructed the ancestral sequences at each node of the phylogenetic trees for each subtype and counted the number of times each mutation was predicted to have developed within a subtype. Mutations for which founder viruses accounted for 75% or more of occurrences (i.e., the number of branches on which the mutation has developed divided by the number of sequences with the mutation is less than 75%) were not considered treatment-related mutations. For this analysis, phylogenetic trees were created using the neighbor-joining method using the HKY85 model with gamma distribution within PAUP^*^ version 4.0b10 for each subtype and each gene. Ancestral sequences were reconstructed using MESQUITE version 1.02 (http://www.mesquiteproject.org).

To further reduce the influence of transmitted drug resistance on this analysis and to exclude the possibility that some untreated persons were classified incorrectly, sequences from untreated persons containing two or more non-polymorphic resistance mutations were excluded from the analysis. This approach was predicated on the strong likelihood that the presence of two mutations at highly conserved sites in untreated persons does not reflect natural variation in protease or RT but rather is most consistent with previous selective drug pressure. Based on this criterion, 23 protease and 25 RT sequences from 47 persons were excluded from the analysis: 22 CRF01_AE sequences, 15 subtype C sequences, six CRF02_AG sequences, five subtype G sequences, four subtype A sequences, three subtype F sequences, and one subtype D sequence. The mutations in the excluded sequences consisted almost entirely of the NRTI-resistance mutations at positions 41, 67, 70, 210, 215, and 219; the NNRTI-resistance mutations at positions 103, 181, and 190; and the PI-resistance mutations at positions 48, 82, and 90. An analysis that included these 56 sequences did not affect any of the significant findings in the study because these mutations were so much more common in treated than in untreated persons in multiple different subtypes (data not shown).

### Statistical Analysis

**Figure 1:**
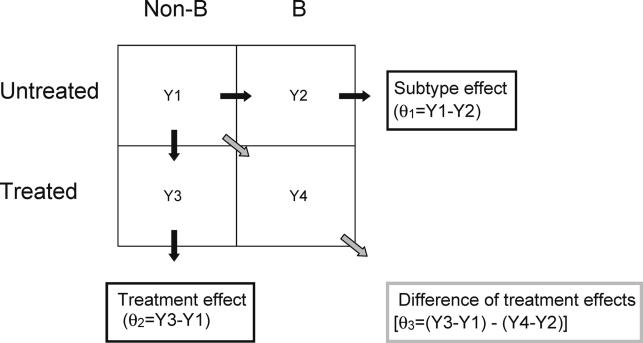
Binomial Response Model Used to Evaluate Subtype and Treatment Effects on Genotypic Evolution for Each Protease and RT Position A separate model was created for each non-B subtype. The frequencies of mutations at each position in four patient groups (untreated subtype B, treated subtype B, untreated non-B, and treated non-B) were converted to *Y* scores using a cube root transformation (similar to a logistic transform). Subtype effect was evaluated by calculating θ_1,_ the score differences between non-B and B subtypes in untreated persons_._ The treatment effect was evaluated by calculating θ_2,_ the score differences between treated and untreated persons within the same subtype. The subtype–treatment interaction was evaluated by calculating θ_3_, the difference of differences in the 2 × 2 table, or the difference in treatment effects between non-B and B subtypes.

Frequencies of mutations at each RT and protease codon were analyzed by a binomial response model employing a cube root transformation (similar to a logistic transform, with higher accuracy for extreme values) to identify significant differences in polymorphisms and treatment-related mutations between subtypes. Mutation frequencies for treated and untreated persons were compared for each subtype (Figure 1). This analysis defined three parameters: (i) a subtype parameter (θ_1_), comparing codons between untreated persons infected with B and each of the non-B subtypes; (ii) a treatment parameter (θ_2_), comparing codons between treated and untreated sequences of the same subtype; and (iii) an interaction parameter (θ_3_), comparing the effect of treatment between subtype B and each of the non-B subtypes.

To increase the statistical power of our analysis, we made two simplifications. First, we did not distinguish between distinct substitutions at the same position; all differences from consensus B were considered mutations. Second, viruses were categorized only according to the classes of drugs (PI, NRTI, NNRTI) to which they had been exposed.

To correct for multiple comparisons between subtype B and each non-B subtype, significant results included those *z* values exceeding three in absolute value, according to a 0.05 Benjamini–Hochberg false discovery rate criterion [41]. This method is a sequential Bonferroni-type procedure that is appropriate for situations in which multiple statistically significant associations are expected. The coefficients in the binomial response model were ranked in ascending order, and each hypothesis of rank *r* was compared with a significance cutoff of 0.05 (false discovery rate) multiplied by *r/n,* where *n* was 99 for the protease mutations and 240 for the RT mutations (i.e., number of comparisons).

## Results

### HIV-1 Subtypes

**Figure 2:**
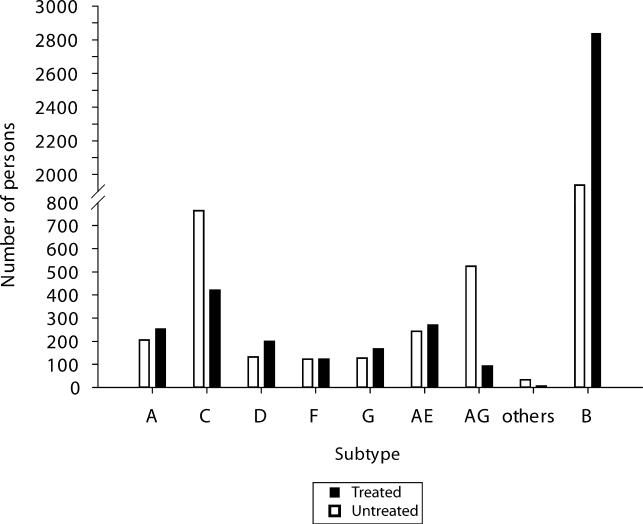
Number of Treated and Untreated Persons Infected with B and Non-B HIV-1 Subtypes from Whom Protease and/or RT Sequences Were Available for Analysis

Sequences were obtained from 3,686 persons, in 56 countries, infected with non-B HIV-1 subtypes (Figure 2; Table 1), including 1,997 persons from whom both protease and RT sequences were available, 908 persons from whom only protease sequences were available, and 933 persons from whom only RT sequences were available. Sequences from untreated individuals were isolated between 1983 and 2003. Sequences from treated individuals were isolated between 1993 and 2003. A total of 2,311 (82%) protease and 2,381 (83%) RT sequences were obtained from plasma samples. The remaining sequences were obtained from peripheral blood mononuclear cells.

**Table 1:**
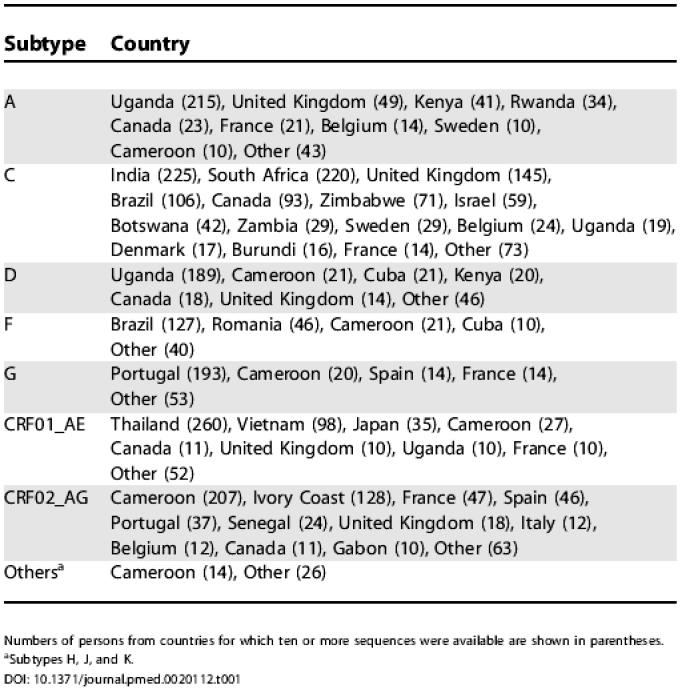
Geographical Origin of Persons Infected with Non-B Subtypes

Numbers of persons from countries for which ten or more sequences were available are shown in parentheses. ^a^Subtypes H, J, and K. DOI: 10.1371/journal.pmed.0020112.t001

### Antiretroviral Treatments

**Figure 3:**
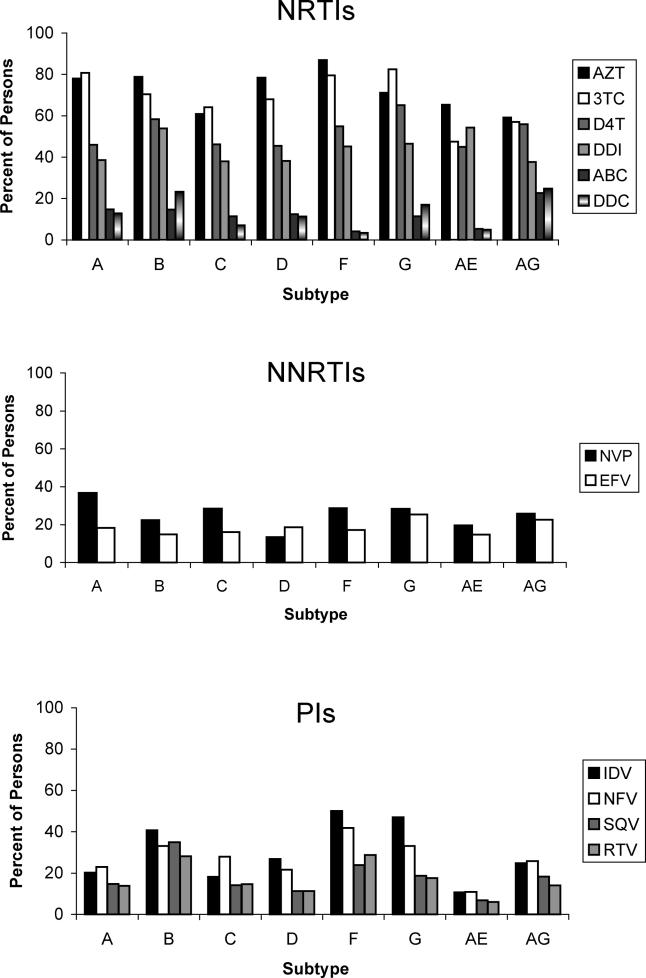
Proportions of Persons Receiving Treatment with Specific NRTIs, NNRTIs, and PIs The number of persons with non-B virus receiving the NRTI tenofovir (ten), the NNRTI delavirdine (three), and the PIs amprenavir (13) and lopinavir (28) are not shown. 3TC, lamivudine; ABC, abacavir; AZT, zidovudine; D4T, stavudine; DDC, zalcitabine; DDI, didanosine; EFV, efavirenz; IDV, indinavir; NFV, nelfinavir; NVP, nevirapine; RTV, ritonavir; SQV, saquinavir.

Of the participants with non-subtype-B viruses, 1,533 (42%) were receiving antiretroviral drugs at the time of sequencing: 1,140 were receiving NRTIs, 527 PIs, and 766 NNRTIs. According to subtype, 89% to 100% had received one or more NRTIs, 22% to 76% had received one or more PIs, and 32% to 55% had received one or more NNRTIs. Among treated persons infected with subtype B viruses, 98% had received NRTIs, 66% had received PIs, and 34% had received NNRTIs (Figure 3).

### Mutation Prevalence

Twenty-two (22%) protease and 87 (36%) RT positions were conserved in all subtypes regardless of the presence or absence of therapy. Twenty-four (24%) protease and 38 (16%) RT positions were conserved in untreated persons but were mutant in more than 1% of treated persons. The remaining 53 (53%) protease and 115 (48%) RT positions were polymorphic, or mutant in more than 1% of untreated persons.

To assess the impact of viral subtype and treatment on the distribution of mutations in protease and RT, a binomial response model using subtype and treatment as explanatory variables was used to predict whether a position was wild-type (matching the consensus B site) or mutant. This model identified three types of positions: (i) positions in sequences from untreated people more likely to be mutated in non-B than in B subtypes (subtype-specific polymorphisms); (ii) positions in sequences of the same subtype more likely to be mutated in treated than in untreated people (subtype-specific treatment-related positions); and (iii) positions for which the effect of treatment differed significantly between non-B and B subtypes (subtype–treatment interactions).

#### Subtype-specific polymorphisms

**Figure 4:**
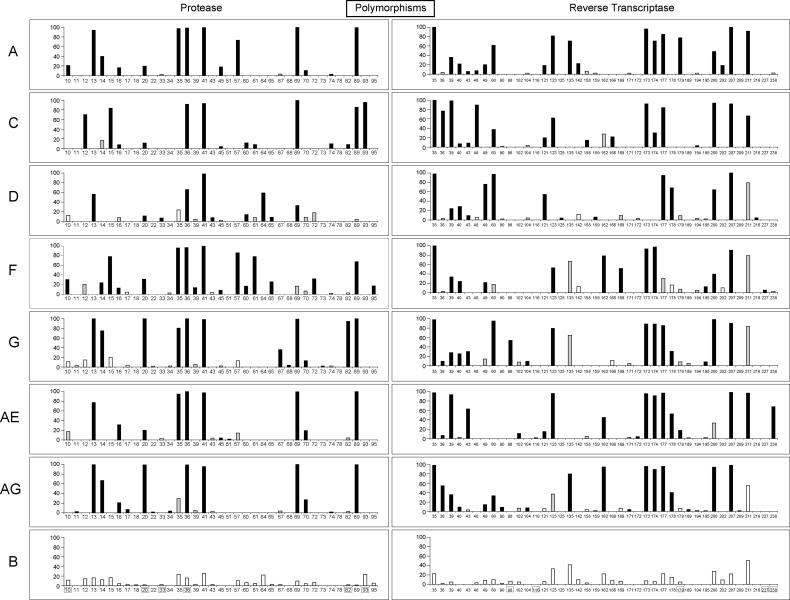
Subtype-Specific Polymorphisms Positions in protease (left) and RT (right) at which mutation frequency varied significantly between subtype B and at least one non-B subtype in untreated persons. Positions are shown along the x-axes, and the frequency of mutation for each subtype is shown along the y-axes. Positions related to drug resistance in subtype B are boxed. Bar colors denote statistical significance: black is statistically significant (Z_θ1_ ≥ 3); gray is borderline significant (1 ≤ Z_θ1_ < 3); white is not statistically significant (Z_θ1_ < 1).

Figure 4 shows the mutation prevalence according to subtype for 37 protease and 41 RT subtype-specific polymorphisms (significant θ_1_; see Methods). Twenty-eight of the protease and 26 of the RT subtype-specific polymorphisms were polymorphic in untreated subtype B viruses, whereas nine of the protease and 15 of the RT were conserved in subtype B. Subtype-specific polymorphisms at conserved positions in untreated subtype B viruses were generally present in a small number of subtypes at low levels (<5%). Notable exceptions included protease positions 45 and 74 and RT positions 40, 43, 104, 195 and 238.

Six subtype-specific polymorphisms in protease (positions 10, 20, 33, 36, 82, and 93) and five in RT (98, 116, 179, 227, and 238) occurred at sites known to be associated with drug resistance in subtype B viruses. These positions (with the exception of positions 116, 227, and 238 in RT) were also polymorphic in subtype B. M184I was present in six monophyletic CRF01_AE isolates from untreated persons [42,43] and was therefore not considered to be a subtype-specific polymorphism. These six sequences also displayed G→A hypermutation [44], possibly explaining the M184I change (ATG→ATA) and further complicating the significance of this finding.

Subtype-specific polymorphisms at four protease and three RT drug-resistance positions represented the consensus sequence for at least one non-B subtype: K20I in subtypes G and CRF02_AG, M36I in subtypes A, C, D, F, G, CRF01_AE and CRF02_AG, V82I in subtype G, and I93L in subtype C for protease; and A98S in subtype G, V179I in subtype A, and K238R in CRF01_AE for RT. Each of the non-B subtypes was significantly more polymorphic than subtype B at protease positions 20, 36, and 41 and RT positions 35, 39, and 207.

#### Subtype-specific treatment-related mutations.

**Figure 5:**
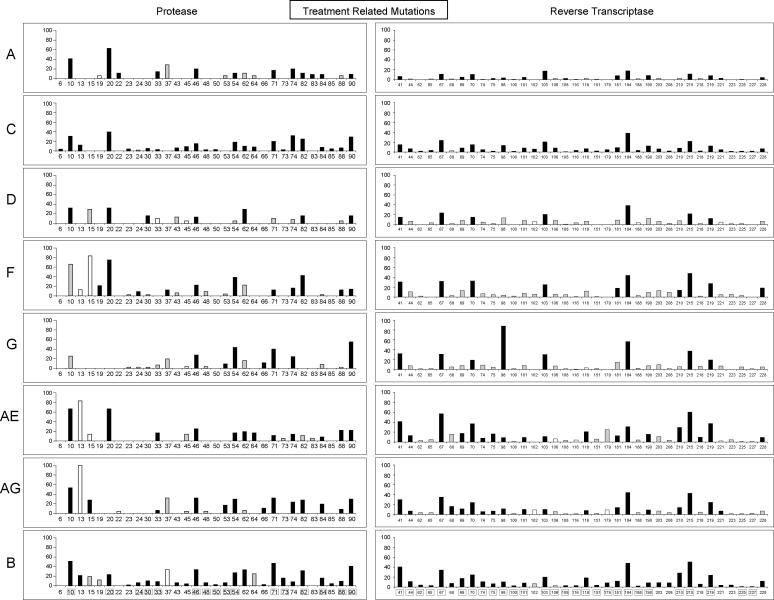
Subtype-Specific Treatment-Related Mutations Positions in protease (left) and RT (right) at which mutations were significantly more prevalent in HIV-1 isolates from treated than from untreated persons infected with the same subtype. Positions are shown along the x-axes, and the proportion of mutant sequences in treated persons for each subtype is shown along the y-axes. For protease (left), treated persons are those receiving one or more PIs. For RT (right), treated persons are those receiving one or more NRTIs. Positions related to drug resistance in subtype B are boxed. Bar colors denote statistical significance: black is statistically significant (Z_θ2_ ≥ 3); gray is borderline significant (1 ≤ Z_θ2_ < 3); white is not statistically significant (Z_θ2_ < 1).

Figure 5 shows the mutation prevalence according to subtype for 31 protease and 36 RT subtype-specific treatment-related positions significantly more likely to be mutant in treated than untreated persons in at least one non-B subtype (significant θ_2_; see Methods). The protease positions included 16 of the 22 known PI resistance positions and 15 additional treatment-related positions. The RT positions included 28 of the known 33 RTI resistance positions and eight additional treatment-related positions. Although each of the known PI- and RTI-resistance positions occurred in at least one non-B subtype, three of the 22 protease positions and five of the 33 RT positions included mutations that occurred too infrequently for a significant association with treatment to be detected in our analysis.

Of the 15 treatment-related protease positions not known to be associated with drug resistance, eight were also significantly associated with treatment in subtype B viruses (positions 13, 23, 43, 45, 62, 66, 74, and 85), and two have been previously reported to be associated with treatment in subtype B viruses (positions 22 and 83) [45]. The remaining five subtype-specific treatment-related protease positions included positions 6, 15, 19, 37 and 64, which—although highly polymorphic in many subtypes—are associated with treatment in subtype C (positions 6 and 64), CRF02_AG (position 15), subtype F (position 19), subtype A (position 37), and CRF01_AE (position 64).

Of the eight treatment-related RT positions at sites not known to be associated with drug resistance, seven were also significantly associated with treatment in subtype B viruses (positions 68, 203, 208, 218, 221, 223, and 228) and one (position 102) was associated with treatment in subtype C but not B.

#### Subtype–treatment interactions

The subtype of the sequence significantly influenced the effect of treatment (significant θ_3_; see Methods) on 20 protease positions (10, 12, 13, 14, 15, 20, 37, 53, 62, 63, 64, 65, 67, 71, 73, 74, 77, 82, 88, and 89) and 11 RT positions (35, 39, 48, 98, 104, 106, 121, 162, 166, 179, and 238). For example, RT position 98 was mutant in 7% of untreated and 16% of treated persons with subtype B viruses (approximately 2-fold difference) and in 1% of untreated and 14% of treated persons with CRF01_AG viruses (14-fold difference). Other positions less likely to be mutated in subtype B than in non-B viruses in response to treatment included protease residues 14 (subtype A); 13 and 64 (subtype C); 37 and 65 (subtype F); 71 (subtype G); 62 and 64 (CRF01_AE); and 15 and 71 (CRF02_AG); and RT residues 35 (subtype A); 98 and 106 (subtype C); 35 and 98 (subtype G); and 98 (CRF02_AG).

At other positions, treatment had a larger effect on subtype B viruses than on one or more non-B subtypes. For example, protease position 20 was mutant in 2% of untreated and 24% of treated persons with subtype B viruses (approximately 12-fold increase with treatment) and in 11% of untreated and 42% of treated persons with subtype C viruses (approximately 4-fold increase with treatment). These positions included protease residues 10, 20, and 63 (subtype A); 20, 53, 63, 74, and 82 (subtype C); 13 and 20 (subtype D); 10, 14, 20, and 77 (subtype F); 20, 67, 73, 82, and 88 (subtype G); 20, 63, 82, and 89 (CRF01_AE); and 20 (CRF02_AG); and RT residues 39 and 179 (subtype A); 35, 48, 121, and 166 (subtype C); 39 (subtype D); 39 (subtype F); 39 and 104 (subtype G); 162 and 238 (CRF01_AE); and 39 (CRF02_AG).

These 31 positions with subtype–treatment interactions included 12 known drug-resistance positions. Of these, seven protease (10, 20, 53, 63, 77, 82, and 88) and two RT (179 and 238) resistance positions were more likely to be mutated in subtype B than in one or more non-B subtypes in response to treatment. One protease position (71) and two RT positions (98 and 106) were more likely to be mutated in one or more non-B subtypes.

### Known Drug-Resistance Mutations

Figure 6 shows the amino acid substitutions present at drug-resistance positions in protease and RT sequences from untreated and treated persons infected with B and non-B subtypes. Fourteen of the 22 known PI-resistance positions occurred in subtype A, 20 in subtype C, 16 in subtype D, 20 in subtype F, 18 in subtype G, 17 in CRF01_AE, and 17 in CRF02_AG. Thirteen of the 18 known NRTI-resistance positions occurred in subtype A, 18 in subtype C, 13 in subtype D, 15 in subtype F, 18 in subtype G, 18 in CRF01_AE, and 16 in CRF02_AG. Ten of the 15 known NNRTI-resistance positions occurred in subtype A, 15 in subtype C, 11 in subtype D, 13 in subtype F, 14 in subtype G, 13 in CRF01_AE, and 12 in CRF02_AG.

**Figure 6 A:**
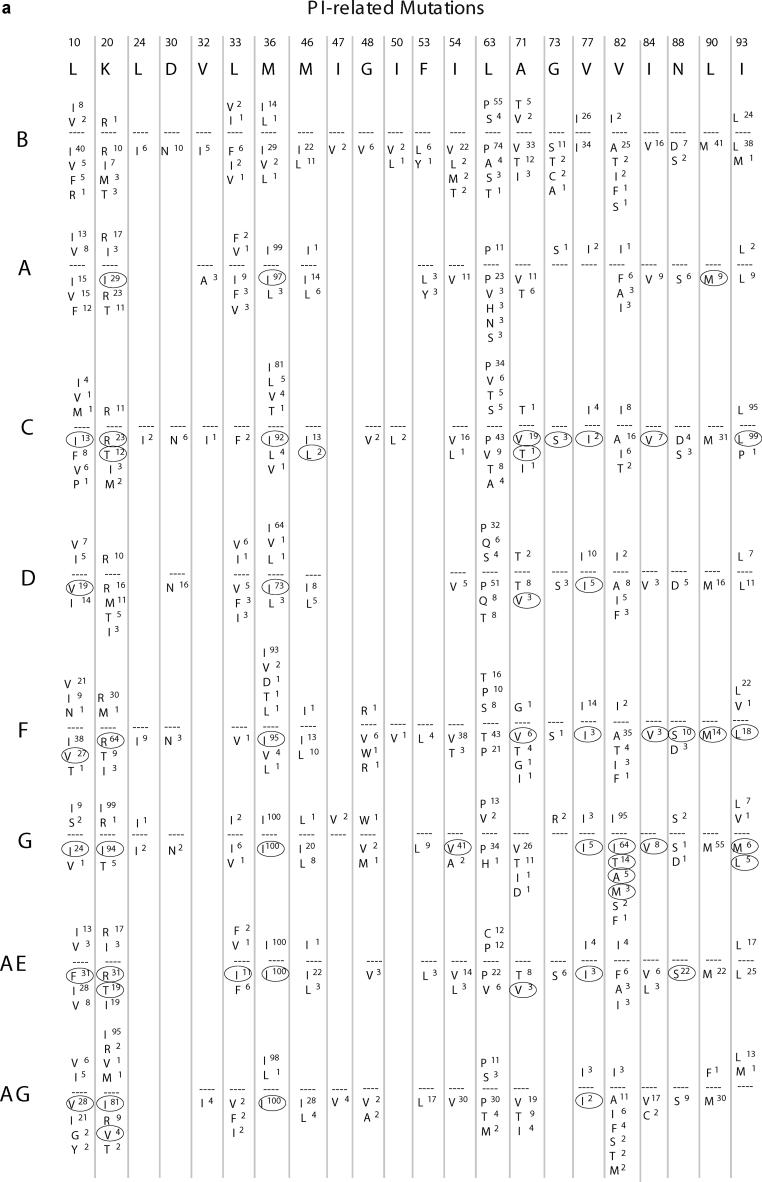
Amino Acid Differences from Consensus B Sequence at Drug-Resistance Positions in Protease and RT according to Subtype (A) shows data for protease, and (B) shows data for RT. In both, the first line lists the drug-resistance positions. The second line shows single-letter amino acid codes for the consensus B sequence. For each subtype (left column), the frequency of specific mutations in untreated persons is shown above the dashed line, whereas the frequency of specific mutations in treated persons is shown below the dashed line. Positions with significant differences in mutation frequency between B and non-B subtypes (*p* < 0.01, according to χ^2^ test with Yate's correction) are circled. A pound sign indicates an insertion.

**Figure 6 B:**
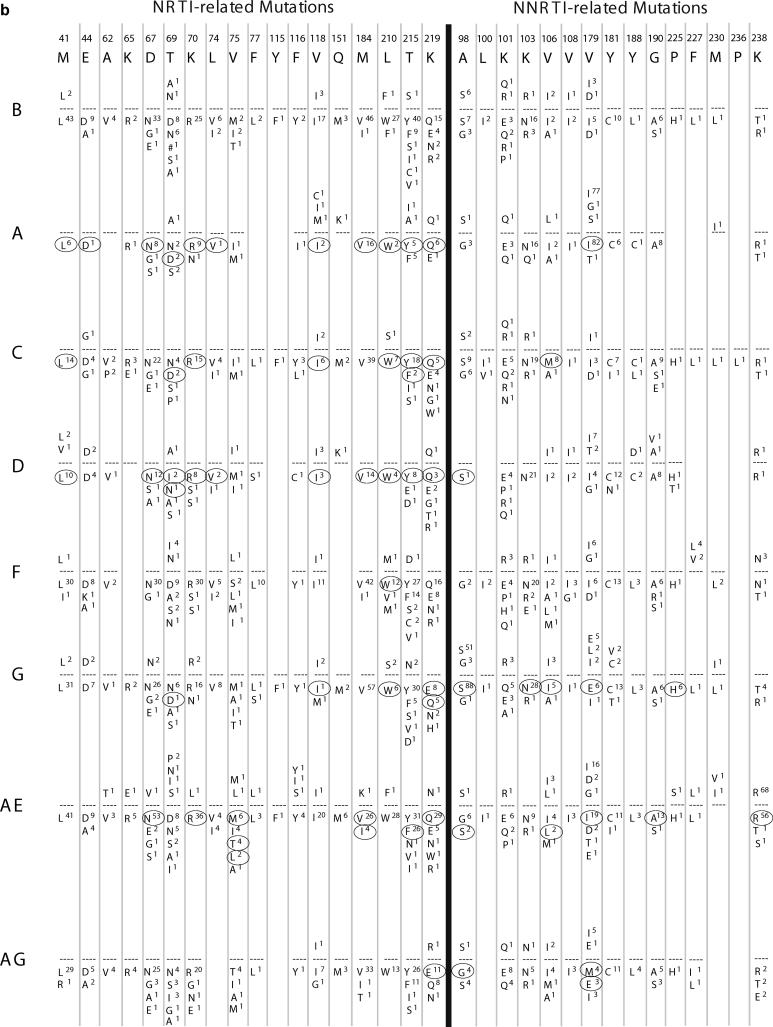
Amino Acid Differences from Consensus B Sequence at Drug-Resistance Positions in Protease and RT according to Subtype (A) shows data for protease, and (B) shows data for RT. In both, the first line lists the drug-resistance positions. The second line shows single-letter amino acid codes for the consensus B sequence. For each subtype (left column), the frequency of specific mutations in untreated persons is shown above the dashed line, whereas the frequency of specific mutations in treated persons is shown below the dashed line. Positions with significant differences in mutation frequency between B and non-B subtypes (*p* < 0.01, according to χ^2^ test with Yate's correction) are circled. A pound sign indicates an insertion.

In all, 106 of 113 (94%) different amino acid substitutions at 55 known subtype B drug-resistance positions (22 protease and 33 RT) were also present in at least one non-B subtype. In an exploratory analysis, which was not controlled for multiple comparisons, the frequencies of 24 mutations at 14 protease positions and 32 mutations at 19 RT positions differed between subtype B and one or more non-B subtypes.

## Discussion

This collaborative analysis was designed to determine whether and to what degree the genetic mechanisms of HIV drug resistance are shared between subtype B and non-B viruses. Mutations responsible for drug resistance in subtype B viruses have been characterized by three types of studies: (i) those that identify mutations selected in viruses of persons receiving antiretroviral therapy, (ii) those that quantify the effect of specific mutations on in vitro drug susceptibility, and (iii) those that examine the effectiveness of treatment regimens in persons with viruses containing known or suspected drug-resistance mutations. This study, which identifies mutations arising in non-B viruses during antiretroviral therapy, is a necessary step for designing laboratory and clinical studies of potential drug-resistance mutations.

Do the known subtype B drug-resistance mutations also occur in non-B subtypes? We found that each of the 55 known subtype B drug-resistance mutations occurred in at least one non-B isolate. Of these, 44 (80%) were significantly associated with drug therapy in non-B isolates. The remaining 11 mutations were uncommon in subtype B and all non-B subtypes, making it difficult to determine whether they were also significantly associated with therapy. Phenotypic susceptibility testing of non-B viruses with treatment-selected mutations is necessary to confirm and quantify the contribution of these mutations to drug resistance in the genetic context in which they arise.

Do non-subtype B viruses from persons with virologic failure develop novel mutations? Fifteen protease and eight RT positions not generally considered to be drug-resistance positions were significantly associated with treatment in at least one non-B subtype. However, mutations at 17 of these 23 positions were also associated with treatment in subtype B viruses. Therefore, of the 67 mutations associated with treatment in at least one non-B subtype, 61 were also associated with treatment in subtype B. For the six mutations associated with therapy in at least one non-B subtype but not in subtype B, the associations were at the borderline of significance and require confirmation.

Among untreated persons, non-B subtype-specific polymorphisms occurred at 37 protease and 41 RT positions. Most of these non-B polymorphic positions are also polymorphic in subtype B viruses, and several act as accessory drug-resistance mutations in subtype B viruses. Phenotypic susceptibility testing of non-B viruses with such polymorphic accessory mutations is needed to confirm that these naturally occurring viruses are fully susceptible to current antiretrovirals—a supposition that appears be true based on the excellent virologic responses of non-B viruses to antiretroviral treatment in observational studies.

We made two simplifications in this study to increase the statistical power of our analyses. These will become unnecessary in future analyses as sufficient numbers of sequences from persons with well characterized treatment histories become available. First, we did not distinguish between different substitutions at the same position; all differences from consensus B were considered mutations. Second, viruses were classified only by the classes of drugs to which they were exposed rather than by individual drugs or drug regimens. Therefore, our analyses could not detect differences between subtype B and other subtypes that depend on specific mutations or specific drugs. Indeed, two such differences have been reported: (i) V106M is the most common substitution at RT position 106 in subtype C viruses whereas V106A predominates in subtype B viruses [33,35,46], and (ii) although the protease mutations D30N and L90M both develop in non-B viruses during nelfinavir therapy, D30N occurs more commonly in subtype B, whereas L90M occurs more commonly in subtypes C, G, and CRF01_AE [17,34,47,48].

Although the clinical samples in this study were originally obtained for a variety of purposes, including clinical management, the sequences of these samples represent experiments of nature that reveal the mutations associated with continued HIV-1 replication in the presence of selective antiretroviral therapy. The accurate identification of treatment-related mutations in such a cross-sectional analysis is challenging, however, because misclassification can result from the transmission of drug-resistant viruses, differences in specific HIV-1 variants among different human populations (population stratification), and the many statistical comparisons required as a result of HIV-1 sequence variability.

The transmission of drug-resistant HIV-1 viruses weakens cross-sectional analyses because some untreated persons may have been infected with viruses already containing treatment-related mutations. To mitigate this effect, we excluded isolates from untreated persons containing two or more non-polymorphic known drug-resistance mutations, because this pattern is not consistent with natural sequence variation. However, as noted in the Methods, an analysis that included these isolates did not alter any of the significant findings in the study. Conversely, the possibility that resistance mutations transmitted between persons in our dataset inflated the amount of resistance among persons receiving treatment was mitigated by excluding any isolate differing from another isolate at less than 1% of its nucleotides.

HIV-1 evolution is driven by genetic drift, immunologic pressure, and selective drug pressure. Population stratification can be a confounding factor when viral lineages with different founder mutations (resulting from drift or immunologic pressure) are exposed to different degrees of antiretroviral selection pressure. To distinguish mutations developing in multiple individuals as a result of selective drug pressure from mutations originating in a fewer number of founder viruses, we reconstructed the ancestral sequences at each node of a phylogenetic tree for each subtype and counted the number of times each mutation was predicted to have developed within that subtype. Because of the limited ability of phylogenetic methods to estimate accurate trees for large numbers of related sequences (i.e., belonging to the same HIV-1 subtype), only those positions for which the majority of mutations (≥75%) appeared to result from new mutations were considered to be selected by antiretroviral therapy.

Because this analysis was, to our knowledge, the first to simultaneously examine all protease and most polymerase-coding RT positions in multiple subtypes, and because multiple associations between mutation and treatment were expected, we used a relatively lenient correction for multiple comparisons in order to minimize the number of missed associations. Nonetheless, of the 67 positive associations detected in this study, 61 were also present in persons with subtype B viruses and have previously been reported [45,49].

In conclusion, most of the protease and RT positions associated with drug resistance in subtype B viruses are selected by antiretroviral therapy in one or more non-B subtypes as well. Conversely, we found no evidence that non-B viruses develop resistance by mutations at positions that are not associated with resistance in subtype B viruses. Based on currently available data, global surveillance efforts and genotypic assessments of drug resistance should focus primarily on the known subtype B drug-resistance mutations.

## Supporting Information

Dataset S1 List of GenBank Accession Numbers for Non-Subtype-B Sequences Used in This Study (59 KB PDF).

### Accession Numbers

The GenBank (http://www.ncbi.nlm.nih.gov/Genbank/) isolates and accession numbers for the reference subtype specimens discussed in this paper are U455/subtype A (M62320), HXB2/subtype B (K03455), C2220/subtype C (U46016), NDK/subtype D (M27323), 93BR020/subtype F (AF005494), SE6165/subtype G (AF061642), 90CR056/subtype H (AF005496), SE9173c/subtype J (AF082394), 97EQTB11C/subtype K (AJ249235), CM240/CRF01_AE (U54771), IbNG/CRF02_AG (L39106), YBF30/Group N (AJ006022), and ANT70C/Group O (L20587). The accession numbers for the non-subtype-B sequences used in this study are listed in Dataset S1.

**Patient Summary**

**Background**

There are many different subtypes of HIV-1. The most common one in more developed countries is subtype B and that is the one which has been studied most and used in drug development. However, worldwide, other subtypes are more frequent. All HIV-1 subtypes acquire mutations, and some of these cause resistance to the drugs used to treat HIV. It is not clear whether the same mutations that cause drug resistance to subtype B virus are also important in causing resistance to non-subtype-B viruses.

**What Did the Researchers Do?**

They compared the viral sequences of 3,686 people with non-subtype-B HIV, and 4,769 with subtype B virus, all with known treatment histories. They found that the mutations known to cause drug resistance in subtype B virus also occur in non-subtype B, and the majority of mutations in non-subtype B also occur in subtype B.

**What Do These Findings Mean?**

It seems that largely the same mutations occur in both subtype B and non-subtype-B viruses. However, some mutations were only present in low numbers, so more work will need to be done before their role is clear. Also, the authors did not look at mutations and their relation to each different drug a patient had—only the general type of drug. Nor did they look at what happens when different mutations occur at one place in a virus. However, for now the current strategy of focusing on assessing the mutations seen in subtype B virus seems a reasonable approach to take when assessing surveillance of drug resistance while more work is done to follow up these findings.

**Where Can I Get More Information?**

TheBody.com has a section on drug resistance: http://www.thebody.com/treat/resistance.html

The Aidsmap Web site has many patient information sheets, including on resistance: http://www.aidsmap.com

## Abbreviations

NRTI: nucleoside reverse transcriptase inhibitor
NNRTI: non-nucleoside reverse transcriptase inhibitor
PI: protease inhibitor
RT: reverse transcriptase
RTI: reverse transcriptase inhibitor
