# Supplementary material for: Impact of HIV-1 Subtype and Antiretroviral Therapy on Protease and Reverse Transcriptase Genotype: Results of a Global Collaboration
Source: PLoS Med. 2005 Apr 26;2(4):e112. doi: 10.1371/journal.pmed.0020112 (PMC1087220; doi:10.1371/journal.pmed.0020112)
Supplement: Dataset S1 — (59 KB PDF). [file pmed.0020112.sd001.pdf]

List of accession numbers for Kantor et al. manuscript

AB078694, AB078705, AB078706, AB078707, AB078713, AB078716, AB081151, AB081153, AB081154, AB081155, AB081156, AB081158, AB081159, AB081160, AB081161, AB081162, AB081163, AB081164, AB081165, AB081166, AB081167, AB081168, AB081169, AB081170, AB081171, AB081172, AB081173, AB081175, AB081176, AB081177, AB081179, AB081180, AB081181, AB081182, AB081184, AB081185, AB081186, AB081187, AB081188, AB081189, AB081190, AB081191, AB081192, AB081193, AB081195, AB081196, AB081197, AB081198, AB081199, AB081200, AB081202, AB081203, AB097866, AB097867, AB097871, AB097872, AF000479, AF000480, AF000481, AF000482, AF000483, AF000484, AF000485, AF000486, AF000487, AF000488, AF000489, AF000490, AF000491, AF000492, AF000493, AF000494, AF000495, AF000517, AF000518, AF000519, AF004885, AF005494, AF005496, AF009382, AF009392, AF009395, AF009396, AF009397, AF009398, AF009399, AF009400, AF009401, AF009402, AF009403, AF009404, AF009405, AF009406, AF009407, AF009408, AF009409, AF009410, AF025721, AF025723, AF025725, AF025727, AF025729, AF025730, AF025733, AF025739, AF025740, AF025741, AF025742, AF025743, AF025745, AF056638, AF061642, AF063223, AF063224, AF067154, AF067155, AF067156, AF067157, AF067159, AF069669, AF069670, AF069671, AF069672, AF069673, AF071473, AF071474, AF075668, AF075669, AF075670, AF075671, AF075672, AF075674, AF075675, AF075677, AF079985, AF079986, AF079987, AF079988, AF079989, AF079995, AF079996, AF083257, AF083258, AF083259, AF083261, AF083262, AF083263, AF083264, AF083265, AF083266, AF083267, AF083268, AF084936, AF125281, AF125283, AF125284, AF125286, AF125287, AF125288, AF125289, AF125291, AF125292, AF125293, AF125294, AF125295, AF136787, AF136788, AF136790, AF136795, AF136796, AF136802, AF165581, AF165583, AF165584, AF165585, AF170545, AF170548, AF176038, AF177347, AF177348, AF177350, AF177351, AF177352, AF177353, AF177354, AF177355, AF177356, AF177358, AF177359, AF177360, AF177362, AF177363, AF177364, AF177365, AF177366, AF177367, AF177368, AF177369, AF177370, AF177371, AF177372, AF177373, AF177374, AF177375, AF177376, AF177544, AF180667, AF180669, AF180670, AF180671, AF184155, AF187037, AF187038, AF187039, AF188335, AF188336, AF188338, AF188339, AF188341, AF188342, AF188343, AF188345, AF188346, AF188347, AF188349, AF188350, AF188494, AF188495, AF188497, AF188498, AF188499, AF188500, AF188501, AF188502, AF188503, AF191189, AF191192, AF191196, AF197340, AF197341, AF199237, AF199239, AF199275, AF199276, AF199278, AF199280, AF199284, AF199286, AF199287, AF199291, AF199293, AF199295, AF199297, AF199299, AF199303, AF199305, AF199307, AF199311, AF199313, AF199315, AF199317, AF216978, AF216979, AF216980, AF216981, AF216982, AF216983, AF216984, AF216985, AF216986, AF216987, AF216988, AF216989, AF216991, AF216993, AF216994, AF216995, AF216996, AF216997, AF216998, AF216999, AF221037, AF247018, AF247019, AF247020, AF247021, AF247022, AF247023, AF247024, AF247028, AF247036, AF247037, AF252045, AF252092, AF252132, AF252133, AF268325, AF268326, AF268329, AF268330, AF268331, AF268332, AF268333, AF268335, AF268336, AF268337, AF268339, AF273161,

AF273211, AF286224, AF286225, AF286227, AF286231, AF286232, AF286234, AF286235, AF288805, AF289548, AF289550, AF295281, AF295282, AF295283, AF295284, AF295285, AF295287, AF295288, AF295289, AF295290, AF295292, AF295293, AF295294, AF295295, AF295296, AF295298, AF295299, AF315754, AF315768, AF326173, AF326174, AF326180, AF326184, AF326189, AF326190, AF326192, AF326195, AF326198, AF328563, AF328565, AF328570, AF328572, AF328573, AF328575, AF328579, AF328586, AF328587, AF328589, AF328591, AF328593, AF328594, AF331680, AF331681, AF331685, AF331694, AF331695, AF331699, AF338977, AF338979, AF338984, AF338985, AF338986, AF338990, AF338992, AF338994, AF338997, AF338999, AF339000, AF339009, AF339013, AF339017, AF339020, AF354001, AF354003, AF354004, AF354005, AF354006, AF354008, AF354009, AF354010, AF354018, AF354019, AF354020, AF354021, AF354023, AF354024, AF354025, AF354026, AF354027, AF354028, AF354030, AF354032, AF354034, AF354035, AF354037, AF354038, AF354040, AF354041, AF354043, AF355299, AF355300, AF355301, AF355302, AF355303, AF355304, AF355306, AF355307, AF355308, AF355309, AF355310, AF355311, AF355312, AF355313, AF355315, AF355316, AF355317, AF357631, AF357660, AF358746, AF358747, AF358748, AF358749, AF358750, AF358751, AF358752, AF358753, AF358754, AF358755, AF358756, AF358757, AF358758, AF358759, AF358760, AF361871, AF361872, AF361873, AF361874, AF361875, AF361876, AF361877, AF361878, AF361879, AF368811, AF368812, AF368813, AF368814, AF368815, AF368817, AF368819, AF368821, AF368822, AF368823, AF368827, AF368828, AF368830, AF368831, AF368833, AF368834, AF368835, AF368836, AF368837, AF368838, AF368839, AF368840, AF377954, AF377955, AF377956, AF377957, AF377958, AF377959, AF385356, AF385936, AF388065, AF388066, AF388067, AF388068, AF388069, AF388070, AF388071, AF388072, AF388073, AF388074, AF388075, AF388076, AF388077, AF388078, AF388079, AF388080, AF388081, AF388082, AF388083, AF388084, AF388085, AF388086, AF388087, AF388088, AF388089, AF388090, AF388091, AF388092, AF388093, AF388094, AF388095, AF388096, AF388097, AF388098, AF388099, AF388100, AF388101, AF388102, AF388103, AF388104, AF388105, AF388106, AF388107, AF388108, AF388109, AF388110, AF388111, AF388112, AF388113, AF388114, AF388115, AF388116, AF388117, AF388118, AF388119, AF388120, AF388121, AF388122, AF388124, AF388125, AF388126, AF388127, AF388129, AF388130, AF388131, AF388132, AF388133, AF388134, AF388135, AF388136, AF388137, AF388138, AF388139, AF388141, AF388142, AF388143, AF388144, AF388145, AF388146, AF388147, AF388148, AF388149, AF388150, AF388151, AF388152, AF388153, AF388154, AF388155, AF388156, AF388157, AF388158, AF388159, AF388160, AF388161, AF388162, AF388163, AF388164, AF388165, AF388166, AF400677, AF400679, AF400680, AF406708, AF406709, AF406710, AF406711, AF406712, AF406713, AF406714, AF406715, AF406716, AF406717, AF406719, AF406720, AF406721, AF406722, AF406723, AF406724, AF406725, AF406726, AF406728, AF406730, AF406731, AF411964, AF411965, AF411966, AF411967, AF413832, AF413858, AF413901, AF413906, AF425385, AF425388, AF425389, AF425391, AF425394, AF425395, AF425396, AF425397, AF425398, AF425401, AF425404, AF425410, AF425411, AF425420, AF425421, AF425426, AF425427, AF425432, AF425435,

AF425436, AF425437, AF425438, AF425441, AF425442, AF425443, AF425445, AF425446, AF425447, AF425448, AF425449, AF425450, AF425452, AF425453, AF425454, AF425455, AF425456, AF425457, AF425459, AF425461, AF425462, AF425466, AF425468, AF425469, AF425475, AF425476, AF425484, AF425485, AF425486, AF443074, AF443075, AF443076, AF443077, AF443078, AF443079, AF443080, AF443081, AF443082, AF443083, AF443084, AF443085, AF443086, AF443087, AF443088, AF443089, AF443090, AF443091, AF443092, AF443093, AF443094, AF443095, AF443096, AF443097, AF443098, AF443099, AF443100, AF443101, AF443102, AF443103, AF443104, AF443105, AF443106, AF443107, AF443108, AF443109, AF443110, AF443111, AF443112, AF443113, AF443114, AF443115, AF447815, AF447817, AF447818, AF447822, AF447827, AF447831, AF447832, AF447833, AF447836, AF447839, AF447840, AF447841, AF447842, AF447844, AF447845, AF447846, AF447850, AF447852, AF447853, AF447856, AF447857, AF447858, AF450096, AF450097, AF455606, AF455607, AF455609, AF455613, AF455618, AF455621, AF455622, AF455623, AF455635, AF455636, AF455639, AF455640, AF455644, AF455645, AF455649, AF455657, AF455659, AF455661, AF455665, AF455667, AF457051, AF457052, AF457053, AF457054, AF457055, AF457056, AF457057, AF457058, AF457060, AF457061, AF457062, AF457063, AF457064, AF457065, AF457066, AF457067, AF457068, AF457069, AF457070, AF457071, AF457072, AF457073, AF457074, AF457075, AF457076, AF457077, AF457078, AF457079, AF457080, AF457081, AF457083, AF457084, AF457085, AF457086, AF457087, AF457088, AF457089, AF457090, AF457091, AF466244, AF466718, AF468457, AF468459, AF468460, AF468461, AF468462, AF468463, AF468464, AF468465, AF468466, AF468467, AF468468, AF468469, AF468471, AF468472, AF468473, AF468474, AF468475, AF468476, AF468477, AF468478, AF468479, AF468480, AF468481, AF468483, AF468484, AF468485, AF468486, AF468487, AF468488, AF468489, AF468490, AF468491, AF468492, AF468493, AF479601, AF479611, AF480802, AF480821, AF480822, AF480823, AF480825, AF480829, AF480831, AF480832, AF484477, AF484478, AF484479, AF484480, AF484481, AF484483, AF484485, AF484486, AF484488, AF484490, AF484491, AF484493, AF484494, AF484495, AF484497, AF484498, AF484499, AF484500, AF484501, AF484502, AF484503, AF484506, AF484507, AF484508, AF484509, AF484510, AF484511, AF484512, AF484513, AF484514, AF484515, AF484516, AF484517, AF484518, AF484519, AF484520, AF484521, AF484522, AF504552, AF504555, AF504560, AF504569, AF504578, AF504581, AF504585, AF504586, AF504594, AF504606, AF504614, AF504620, AF504621, AF504623, AF504632, AF504634, AF504638, AF508283, AF508286, AF508288, AF508290, AF517281, AF517285, AF517308, AF527181, AF527186, AF527190, AF527191, AF527193, AF527208, AF527209, AF527213, AF527225, AF527227, AF527232, AF527235, AF527272, AF527273, AF527276, AF527298, AF527317, AF527318, AF527326, AF527327, AF527330, AF527340, AF527341, AF527342, AF527346, AF527349, AF527350, AF527352, AF527353, AF527354, AF527360, AF527361, AF527363, AF543980, AF543981, AF543982, AF543983, AF543984, AF543985, AF543986, AF543987, AF543988, AF543989, AF543990, AF543991, AF543992, AF543993, AF543995, AF543997, AF543998, AF543999, AF544001, AF544002, AF544004, AF544006, AF544009, AJ003212, AJ012427, AJ239233, AJ239243,

AJ249236, AJ249237, AJ249238, AJ249239, AJ251057, AJ276595, AJ276596, AJ279628, AJ279645, AJ279647, AJ279653, AJ286133, AJ286134, AJ286135, AJ286136, AJ286137, AJ286138, AJ286139, AJ286140, AJ286141, AJ286930, AJ286931, AJ286932, AJ286933, AJ286934, AJ286935, AJ286936, AJ286937, AJ286938, AJ286939, AJ286940, AJ286941, AJ286942, AJ286943, AJ286944, AJ286945, AJ286946, AJ286947, AJ286948, AJ286949, AJ286950, AJ286951, AJ286952, AJ286953, AJ286954, AJ286955, AJ286956, AJ286957, AJ286958, AJ286959, AJ286960, AJ286961, AJ286962, AJ286963, AJ286964, AJ286965, AJ286967, AJ286968, AJ286969, AJ286971, AJ286972, AJ286973, AJ286974, AJ286975, AJ286976, AJ286977, AJ286980, AJ286981, AJ286982, AJ286983, AJ286984, AJ286985, AJ286986, AJ286987, AJ286988, AJ286989, AJ286990, AJ286991, AJ286992, AJ286993, AJ286994, AJ286995, AJ286996, AJ286997, AJ286998, AJ286999, AJ287000, AJ287001, AJ287002, AJ287003, AJ287005, AJ287011, AJ287015, AJ287016, AJ287017, AJ287018, AJ287019, AJ287032, AJ287033, AJ287034, AJ287035, AJ287036, AJ287037, AJ287038, AJ287039, AJ287041, AJ287042, AJ287053, AJ287054, AJ313391, AJ313392, AJ313393, AJ313395, AJ313396, AJ313397, AJ313398, AJ313399, AJ313400, AJ313401, AJ313402, AJ313403, AJ313404, AJ313406, AJ313407, AJ313409, AJ313413, AJ313417, AJ313419, AJ412931, AJ412932, AJ412933, AJ412934, AJ412935, AJ412936, AJ412937, AJ412938, AJ412939, AJ412940, AJ412941, AJ412942, AJ412943, AJ412944, AJ412945, AJ412946, AJ412947, AJ412948, AJ412949, AJ412950, AJ412952, AJ412953, AJ412954, AJ412955, AJ412956, AJ412957, AJ412958, AJ412959, AJ412960, AJ412961, AJ412962, AJ412963, AJ412964, AJ412965, AJ412966, AJ412967, AJ412968, AJ412969, AJ412971, AJ412972, AJ412983, AJ412984, AJ412985, AJ412986, AJ412987, AJ412988, AJ412989, AJ412990, AJ412991, AJ412992, AJ412993, AJ412994, AJ412995, AJ412996, AJ412997, AJ412998, AJ412999, AJ413000, AJ413001, AJ413002, AJ413003, AJ413004, AJ413005, AJ413006, AJ413007, AJ413008, AJ413009, AJ413010, AJ413011, AJ413012, AJ413013, AJ413014, AJ413015, AJ413016, AJ413017, AJ413018, AJ413019, AJ413020, AJ413021, AJ413022, AJ413024, AJ413025, AJ413036, AJ419430, AJ419441, AJ419445, AJ419446, AJ419449, AJ419450, AJ419451, AJ419452, AJ419454, AJ419455, AJ419459, AJ419462, AJ419463, AJ419466, AJ419471, AJ419472, AJ419474, AJ419476, AJ419477, AJ419484, AJ419487, AJ419493, AJ419494, AJ419495, AJ419498, AJ419502, AJ419507, AJ419509, AJ419514, AJ419516, AJ419517, AJ419518, AJ419519, AJ534987, AJ534988, AJ534990, AJ534991, AJ534992, AJ534993, AJ534994, AJ534999, AJ535002, AJ535006, AJ535007, AJ535012, AJ535013, AJ535015, AJ535016, AJ535019, AJ535020, AJ535021, AJ535025, AJ535028, AJ535029, AJ535030, AJ535034, AJ535037, AJ535039, AJ535040, AJ577727, AJ577729, AJ577734, AJ577740, AJ577746, AJ577864, AJ577867, AJ577870, AJ577879, AJ577882, AJ577884, AJ577887, AJ577889, AJ577901, AJ577902, AJ577904, AJ577905, AJ577911, AJ577912, AJ577913, AJ577914, AJ577918, AJ577919, AJ577920, AJ577921, AJ577937, AJ577940, AJ577944, AJ577959, AJ577965, AJ577966, AJ577968, AJ577973, AJ577974, AJ577978, AJ577983, AJ577987, AJ577990, AJ577992, AJ578156, AJ578163, AJ578169, AJ578173, AJ578176, AJ578177, AJ578178, AJ583710, AJ583711, AJ583712, AJ583713, AJ583714, AJ583715,

AJ583716, AJ583717, AJ583718, AJ583720, AJ583721, AJ583722, AJ583723, AJ583724, AJ583725, AJ583726, AJ583727, AJ583728, AJ583729, AJ583730, AJ583731, AJ583732, AJ583733, AJ583734, AJ583735, AJ583736, AJ583737, AJ583739, AJ583741, AJ583742, AJ583746, AJ583748, AJ583749, AJ583750, AJ583751, AJ583752, AJ583753, AY007730, AY007731, AY007732, AY007733, AY007734, AY007735, AY007736, AY007737, AY007738, AY007739, AY007740, AY007741, AY007742, AY007743, AY007744, AY007745, AY007747, AY007748, AY007749, AY007750, AY007751, AY007752, AY007753, AY007754, AY007755, AY007756, AY007757, AY007758, AY007759, AY007760, AY007761, AY007762, AY007763, AY007765, AY010371, AY010375, AY010377, AY010380, AY010381, AY010382, AY010386, AY010391, AY010392, AY010393, AY010395, AY010396, AY010398, AY010399, AY010400, AY010402, AY010403, AY010404, AY010407, AY010408, AY010411, AY010413, AY010414, AY010416, AY010418, AY010419, AY010422, AY010427, AY010429, AY010432, AY010433, AY010434, AY010435, AY010438, AY010448, AY010449, AY010450, AY010452, AY010454, AY010458, AY010460, AY010461, AY010465, AY010469, AY010472, AY010476, AY010477, AY010480, AY010485, AY010488, AY010491, AY010495, AY010497, AY010500, AY010501, AY010502, AY010503, AY017010, AY017452, AY017453, AY017454, AY017455, AY017456, AY017457, AY017461, AY017462, AY017464, AY017465, AY017466, AY017467, AY017468, AY017471, AY032091, AY037273, AY037275, AY037276, AY037277, AY037279, AY037280, AY037281, AY043173, AY043174, AY043175, AY043176, AY049532, AY049533, AY049600, AY049601, AY049627, AY049646, AY049647, AY049648, AY049649, AY049651, AY049666, AY049667, AY049672, AY049673, AY049678, AY049679, AY051199, AY051200, AY051201, AY051202, AY051203, AY051204, AY051205, AY051206, AY051207, AY051208, AY051209, AY051210, AY051212, AY051215, AY051219, AY051220, AY051221, AY051222, AY051223, AY051224, AY051225, AY051226, AY051227, AY051229, AY051230, AY051231, AY051232, AY051233, AY051236, AY051237, AY051238, AY051239, AY051240, AY051241, AY051242, AY051243, AY051244, AY051245, AY051246, AY051247, AY051248, AY051249, AY051250, AY051251, AY051252, AY051253, AY051254, AY051255, AY051257, AY051258, AY051259, AY051260, AY051261, AY051262, AY051263, AY051264, AY051265, AY051266, AY051267, AY051268, AY051270, AY051271, AY051272, AY051273, AY051275, AY051276, AY051277, AY051278, AY051279, AY051280, AY090840, AY090841, AY090842, AY090843, AY090844, AY090845, AY090846, AY090847, AY090848, AY090849, AY090850, AY090851, AY090852, AY090853, AY090854, AY090855, AY090856, AY090857, AY090858, AY090859, AY090860, AY136957, AY136958, AY136959, AY136960, AY136961, AY136962, AY136963, AY136964, AY136965, AY136966, AY136967, AY136968, AY136969, AY136971, AY136972, AY136973, AY136975, AY136976, AY136977, AY136978, AY136979, AY136980, AY136981, AY136982, AY136983, AY136984, AY136985, AY136986, AY136987, AY136988, AY136989, AY136990, AY136991, AY136992, AY136993, AY136994, AY136995, AY136996, AY136997, AY136998, AY136999, AY137000, AY137001, AY137002, AY137003, AY137004, AY137005, AY137006, AY137007, AY137008, AY145811, AY145813, AY145817, AY145818, AY145819, AY145822, AY145824, AY145825, AY145826, AY145827, AY145828, AY145829, AY145830, AY162223, AY162224, AY162225,

AY165187, AY165196, AY165205, AY165206, AY165207, AY165208, AY165209, AY165211, AY165212, AY165213, AY165214, AY165215, AY165216, AY165218, AY165219, AY165220, AY165221, AY165223, AY165224, AY165225, AY165226, AY165230, AY165235, AY165239, AY165240, AY165243, AY165249, AY165252, AY165253, AY165255, AY165257, AY165260, AY165263, AY165265, AY165266, AY165272, AY165276, AY165279, AY165281, AY165283, AY173957, AY173958, AY196498, AY196499, AY196500, AY196501, AY196502, AY196503, AY196504, AY196505, AY196506, AY196507, AY196508, AY196509, AY196510, AY196511, AY196512, AY196513, AY196514, AY196515, AY196516, AY196517, AY204310, AY204311, AY204312, AY204313, AY204314, AY204315, AY204316, AY204317, AY204319, AY204320, AY204321, AY204322, AY204324, AY204326, AY204327, AY204328, AY204329, AY204331, AY204332, AY204334, AY204335, AY204336, AY204337, AY204338, AY204340, AY204341, AY204342, AY204344, AY204345, AY204347, AY204348, AY204349, AY207654, AY207655, AY207656, AY207658, AY207659, AY207660, AY207662, AY207663, AY207664, AY207665, AY207666, AY207667, AY207669, AY207670, AY207671, AY207672, AY207673, AY207674, AY207675, AY207676, AY207677, AY207678, AY207679, AY207680, AY207681, AY207682, AY207683, AY207684, AY207685, AY207686, AY207687, AY207688, AY207689, AY207690, AY207691, AY207692, AY207693, AY207694, AY207695, AY207696, AY207697, AY207698, AY207699, AY207700, AY207701, AY207702, AY207703, AY207704, AY207705, AY207706, AY207707, AY207708, AY207709, AY207710, AY207711, AY207712, AY207713, AY207715, AY207716, AY207717, AY207718, AY207719, AY207720, AY207722, AY207723, AY207724, AY207725, AY207726, AY207727, AY207728, AY207729, AY207730, AY207731, AY207732, AY207733, AY207734, AY207737, AY207738, AY207739, AY207740, AY207741, AY207742, AY207743, AY207744, AY207745, AY207746, AY207747, AY207748, AY207749, AY207751, AY207752, AY207753, AY207844, AY207845, AY207846, AY207847, AY207848, AY207849, AY207850, AY207851, AY207852, AY207853, AY207854, AY207855, AY207856, AY207858, AY207859, AY207860, AY207861, AY207862, AY207863, AY207864, AY207865, AY207866, AY207867, AY207868, AY207869, AY207870, AY207871, AY207872, AY207873, AY207874, AY207875, AY207876, AY207877, AY207878, AY207879, AY207880, AY207881, AY207882, AY207883, AY207884, AY207885, AY207886, AY207887, AY207888, AY207889, AY207890, AY207891, AY207892, AY207893, AY207894, AY207895, AY207896, AY207897, AY207898, AY207900, AY207901, AY207902, AY207903, AY207904, AY207905, AY207906, AY207907, AY207908, AY207909, AY207910, AY207911, AY207913, AY207914, AY207915, AY207916, AY207917, AY207918, AY207919, AY207921, AY207922, AY207923, AY207924, AY207925, AY207926, AY207927, AY207928, AY207929, AY207930, AY207931, AY207932, AY207933, AY207934, AY207935, AY207936, AY207937, AY207938, AY207939, AY213196, AY213200, AY213201, AY213215, AY213220, AY213221, AY213227, AY213239, AY213240, AY213267, AY213277, AY213321, AY213322, AY213326, AY213332, AY213340, AY213351, AY213355, AY213356, AY213360, AY213361, AY213376, AY213377, AY213386, AY213387, AY213402, AY213404, AY213411, AY213413, AY213414, AY213429, AY213435, AY213436, AY213449, AY213460, AY213467, AY213469, AY213470, AY213474, AY213477, AY213480, AY213481, AY213484, AY213488,

AY213489, AY213490, AY213493, AY213508, AY213511, AY213515, AY213518, AY213519, AY213520, AY213521, AY213522, AY213525, AY213534, AY213536, AY213542, AY213544, AY213549, AY213550, AY237961, AY237962, AY237977, AY237979, AY237987, AY237996, AY237997, AY237998, AY238000, AY238006, AY238017, AY238020, AY238028, AY238031, AY238032, AY238047, AY238048, AY238053, AY238054, AY238061, AY238072, AY238080, AY238081, AY238084, AY238085, AY238089, AY238091, AY238093, AY238097, AY238098, AY238099, AY238102, AY238103, AY238104, AY238105, AY238106, AY238109, AY238112, AY238119, AY238123, AY238128, AY238130, AY238132, AY238145, AY238146, AY238147, AY238149, AY238150, AY238152, AY238153, AY238154, AY238155, AY238156, AY238159, AY238162, AY238164, AY238169, AY238176, AY238180, AY238185, AY238186, AY238194, AY238203, AY238204, AY238206, AY238208, AY238211, AY238215, AY238216, AY238221, AY238225, AY238226, AY238228, AY238229, AY238230, AY238233, AY238234, AY238236, AY238237, AY238238, AY238240, AY238244, AY238245, AY238247, AY238249, AY238251, AY238253, AY238254, AY238256, AY238265, AY238268, AY238269, AY238272, AY238274, AY238283, AY238287, AY238289, AY238291, AY238296, AY238299, AY238302, AY238303, AY238306, AY238307, AY238308, AY238310, AY238312, AY238313, AY238314, AY238315, AY242880, AY242920, AY242926, AY260405, AY260406, AY260408, AY260409, AY260411, AY260412, AY260413, AY260414, AY260415, AY260416, AY260417, AY260420, AY260421, AY260422, AY260423, AY260425, AY260426, AY260427, AY260428, AY260429, AY267276, AY267321, AY285115, AY285116, AY285118, AY285119, AY285121, AY285124, AY285125, AY285130, AY285133, AY285136, AY285139, AY285141, AY333452, AY333453, AY333454, AY333456, AY333457, AY333460, AY333461, AY333463, AY333465, AY333471, AY333473, AY333478, AY333484, AY333487, AY333488, AY333491, AY333493, AY333496, AY333498, AY333500, AY333501, AY333503, AY333504, AY333507, AY333509, AY333511, AY333513, AY333516, AY333519, AY333520, AY333521, AY333522, AY333525, AY333529, AY333532, AY333534, AY333540, AY333549, AY333552, AY333562, AY333568, AY333570, AY333571, AY333577, AY333579, AY333583, AY333585, AY333586, AY333587, AY333588, AY333590, AY333596, AY333599, AY333600, AY333601, AY333608, AY358036, AY358037, AY358039, AY358040, AY358041, AY358042, AY358043, AY358044, AY358049, AY358050, AY358051, AY358052, AY358053, AY358054, AY358055, AY358056, AY358057, AY358059, AY358060, AY358061, AY358063, AY358064, AY358067, AY358068, AY358070, AY358071, AY358072, AY359683, AY359685, AY359686, AY359687, AY359688, AY359689, AY359690, AY359692, AY359693, AY359694, AY359695, AY359697, AY359698, AY359699, AY359700, AY359703, AY359704, AY359705, AY359706, AY359707, AY359708, AY359709, AY359711, AY359712, AY359713, AY359714, AY359715, AY359716, AY359717, AY359718, AY359719, AY359720, AY359721, AY359723, AY359724, AY359726, AY359727, AY359728, AY359730, AY359732, AY359734, AY359735, AY359736, AY359737, AY359738, AY359739, AY359740, AY359741, AY359742, AY359743, AY359744, AY359745, AY359747, AY359748, AY359749, AY359750, AY359751, AY359752, AY359753, AY359754, AY359755, AY359756, AY359757, AY359758, AY359759, AY359760, AY359761, AY359762, AY359764, AY359765, AY359766, AY359767, AY359768, AY359769,

AY359770, AY359771, AY359772, AY359775, AY359777, AY359778, AY359779, AY359781, AY359782, AY359783, AY359784, AY359785, AY359786, AY359787, AY359788, AY359789, AY359790, AY359792, AY359793, AY359794, AY359795, AY359797, AY359798, AY359799, AY359801, AY359802, AY359803, AY359804, AY359805, AY359806, AY359807, AY371703, AY371704, AY371705, AY425349, AY425350, AY425351, AY425352, AY425353, AY425354, AY425355, AY425356, AY425357, AY435220, AY435221, AY435222, AY435223, AY435224, AY435225, AY435226, AY435227, AY435228, AY435229, AY435230, AY435231, AY435232, AY435233, AY435234, AY435235, AY435236, AY435237, AY435238, AY435239, AY435240, AY435241, AY435242, AY435243, AY435244, AY435245, AY435246, AY435247, AY435248, AY435250, AY435251, AY435252, AY435253, AY435254, AY435255, AY435256, AY435257, AY435258, AY435259, AY435260, AY435261, AY435263, AY435264, AY435265, AY435266, AY435267, AY435268, AY435269, AY435270, AY435271, AY435272, AY435273, AY435274, AY435275, AY435277, AY435278, AY435279, AY435280, AY435281, AY435282, AY435283, AY435284, AY435285, AY435286, AY435287, AY435288, AY435289, AY435290, AY435292, AY435293, AY435294, AY435295, AY435296, AY435297, AY435298, AY435299, AY435300, AY435301, AY435302, AY435303, AY435304, AY435305, AY435306, AY435307, AY435308, AY435309, AY435310, AY435311, AY435312, AY435313, AY435314, AY435315, AY435316, AY435317, AY435318, AY435319, AY435320, AY435321, AY435322, AY435323, AY435324, AY435325, AY435326, AY435327, AY435328, AY435329, AY435330, AY435331, AY435332, AY435333, AY435334, AY435335, AY435336, AY435337, AY435338, AY435339, AY435340, AY435341, AY435342, AY435343, AY435344, AY435345, AY435346, AY435347, AY435348, AY435349, AY435350, AY435351, AY435352, AY435353, AY435354, AY435355, AY435356, AY435357, AY435358, AY435359, AY435360, AY435361, AY435362, AY435363, AY435364, AY435365, AY435366, AY435367, AY435368, AY435369, AY435370, AY435371, AY435372, AY435373, AY435374, AY435375, AY435376, AY435377, AY435378, AY435379, AY435380, AY435381, AY435382, AY435383, AY435384, AY435385, AY435386, AY435387, AY435388, AY441803, AY441820, AY441826, AY441844, AY441856, AY441873, AY441897, AY441918, AY461491, AY461494, AY461496, AY461501, AY461502, AY492752, AY492753, AY492754, AY492755, AY492756, AY492758, AY492759, AY492760, AY492761, AY492762, AY492763, AY492764, AY492765, AY492766, AY492767, AY492768, AY492769, AY492770, AY492771, AY492772, AY492773, AY492774, AY492775, AY492776, AY492777, AY492779, AY492780, AY492781, AY492783, AY492785, AY492786, AY492787, AY492788, AY492789, AY515728, AY515729, AY515730, AY515731, AY515732, AY515734, AY515735, AY515736, AY515738, AY515740, AY515741, AY515742, AY515743, AY515744, AY515745, AY515746, AY515747, AY515749, AY515750, AY515751, AY515752, AY515753, AY515754, AY515755, AY515756, AY515757, AY515758, AY515759, AY515760, AY515761, AY515762, AY515763, AY515764, AY515765, AY515766, AY515767, AY515768, AY515769, AY515770, AY515771, AY515772, AY515773, AY515774, AY515775, AY515776, AY515777, AY515778, AY515779, AY515780, AY515781, AY515782, AY515783, AY515784, AY515785, AY515786, AY515787, AY515789, AY515790, AY515791, AY515792, AY515793, AY515794, AY515795, AY515796, AY515797, AY515798, AY515799,

AY515800, AY515801, AY515802, AY515803, AY515804, AY515805, AY515806, AY515807, AY515808, AY515809, AY515810, AY515811, AY515812, AY515813, AY515814, AY515815, AY515816, AY515817, AY515818, AY515819, AY515820, AY515821, AY515822, AY515823, AY515824, AY515825, AY515826, AY515827, AY515828, AY515829, AY515830, AY515832, AY515833, AY515834, AY515835, AY515836, AY515837, AY515838, AY515839, AY515840, AY515841, AY515842, AY515843, AY515844, AY515845, AY515846, AY515847, AY515848, AY515849, AY525959, AY525960, AY525961, AY525962, AY525963, AY525964, AY525965, AY525966, AY525967, AY525968, AY525969, AY525971, AY525972, AY525973, AY525974, AY525975, AY525977, AY525978, AY525979, AY525980, AY525981, AY525982, AY525983, AY525984, AY525985, AY525986, AY525988, AY525989, AY525990, AY525991, AY525992, AY525993, AY525995, AY525996, AY525997, AY526000, AY526001, AY526003, AY526004, AY526005, AY526007, AY526008, AY526009, AY526011, AY526012, AY526013, AY526014, AY526015, AY526016, AY526017, AY526018, AY526019, AY526020, AY526022, AY526023, AY526024, AY526025, AY526026, AY526027, AY526028, AY526029, AY526030, AY526031, AY526032, AY526033, AY526034, AY526035, AY526036, AY526037, AY526038, AY526042, AY526043, AY526044, AY526045, AY526046, AY526048, AY526049, AY526050, AY526051, AY526052, AY526053, AY526055, AY526056, AY526058, AY526059, AY526060, AY526062, AY526063, AY526064, AY526065, AY526066, AY526068, AY526069, AY526070, AY526072, AY526074, AY526076, AY526077, AY526078, AY528077, AY529529, AY529530, AY529532, AY529533, AY529534, AY529535, AY529536, AY529538, AY529540, AY529545, AY529547, AY529553, AY529558, AY529560, AY529563, AY529569, AY529572, AY529573, AY529575, AY529576, AY529577, AY529578, AY529579, AY529581, AY529582, AY529583, AY529587, AY529589, AY529590, AY529591, AY529592, AY529593, AY529594, AY529595, AY529596, AY529599, AY529601, AY529602, AY529605, AY529606, AY529607, AY529608, AY529609, AY529610, AY529613, AY529615, AY529616, AY529619, AY529620, AY529621, AY529622, AY556754, AY556755, AY556756, AY556757, AY556758, AY556763, AY556764, AY556766, AY556767, AY556768, AY556770, AY556772, AY556775, AY556777, AY556778, AY556779, AY556780, AY556781, AY556782, AY556784, AY556785, AY556786, AY556788, AY556789, AY556790, AY556791, AY556792, AY556798, AY556799, AY556800, AY556802, AY556803, AY556804, AY556805, AY556806, AY556807, AY556808, AY556809, AY556813, AY556814, AY556815, AY556816, AY556817, AY556818, AY556819, AY556820, AY556822, AY556823, AY556825, AY556826, AY556828, AY556831, AY556832, AY556833, AY556835, AY556836, AY556837, AY556838, AY556839, AY556840, AY556842, AY556843, AY589793, AY589796, AY589798, AY589800, AY589802, AY589804, AY589813, AY589817, AY589818, AY589820, AY589823, AY589826, AY589827, AY589828, AY589830, AY589831, AY589835, AY589836, AY589838, AY589870, AY589872, AY589875, AY589889, AY589890, AY589891, AY589892, AY589893, AY589894, AY589895, AY589896, AY589898, AY589899, AY589900, AY589901, AY589902, AY589903, AY589904, AY589905, AY589906, AY589907, AY589908, AY589909, AY589910, AY589911, AY589912, AY589913, AY589914, AY589915, AY589916, AY589917, AY589918, AY589919, AY589920, AY605547, AY605548, AY605550, AY605551, AY605553, AY605554, AY605555,

AY605556, AY605560, AY605564, AY605567, AY605568, AY605569, AY605570, AY605571, AY605573, AY605574, AY605576, AY605578, AY605579, AY605580, AY605581, AY605582, AY605583, AY605584, AY605586, AY605587, AY605588, AY605589, AY605591, AY605593, AY605594, AY605595, AY605597, AY605598, AY605600, AY605601, AY605602, AY605605, AY605606, AY605607, AY605609, AY605610, AY605611, AY605612, AY605613, AY605614, AY605615, AY605616, AY605617, AY605618, AY605620, AY605621, AY605622, AY605624, AY605629, AY605630, AY605631, AY605633, AY605635, AY605636, AY605639, AY605640, AY605643, AY605645, AY605648, AY643135, AY643141, AY643142, AY643143, AY643145, AY643147, AY643148, AY643149, AY643151, AY643155, AY643158, AY643159, AY643160, AY643161, AY643162, AY643163, AY643166, AY643172, AY643176, AY643177, AY643178, AY643180, AY643181, AY643185, AY643188, AY643201, AY643203, AY643207, AY643208, AY643212, AY643213, AY643214, AY643215, AY643216, AY643218, AY643220, AY643221, AY643222, AY643224, AY643228, AY643230, AY643231, AY643232, AY643233, AY643234, AY643235, AY643236, AY643239, AY643240, AY643245, AY643246, AY643247, AY643249, AY643250, AY643251, AY643260, AY643261, AY643263, AY677418, AY677499, AY677500, AY677502, AY677503, AY677504, AY677505, AY677506, AY677507, AY677508, AY677511, AY677512, AY677513, AY677514, AY677515, AY677516, AY677518, AY677519, AY677520, AY677521, AY677522, AY677523, AY677525, AY677526, AY677528, AY677529, AY677530, AY677532, AY677534, AY677535, AY677536, AY677537, AY677538, AY677539, AY677540, AY677543, AY677545, AY677546, AY677547, AY677548, AY677549, AY677551, AY677552, AY677554, AY677555, AY677556, AY677557, AY677561, AY677563, AY677564, AY677565, AY677566, AY677567, AY677568, AY677571, AY677572, AY677573, AY677574, AY677575, AY677576, AY677577, AY677579, AY677580, AY677581, AY677583, AY677584, AY677585, AY677586, AY677587, AY677588, AY677591, AY677592, AY677593, AY677594, AY677595, AY677596, AY677597, AY677598, AY677599, AY677600, AY677601, AY677602, AY677603, AY677604, AY677605, AY677607, AY677608, AY677610, AY677612, AY677615, AY677616, AY677617, AY677618, AY900671, AY900672, AY900673, AY900674, AY900675, AY900676, AY900677, AY900678, AY900679, AY900680, AY900681, AY900682, AY900683, AY900684, AY900685, AY900686, AY900687, AY900688, AY900689, AY900690, AY900691, AY900692, AY900693, AY900694, AY900695, AY900696, AY900697, AY900698, AY900699, AY900700, AY900701, AY900702, AY900703, AY900704, AY900705, AY900706, AY900707, AY900708, AY900709, AY900710, AY900711, AY900712, AY900713, AY900714, AY900715, AY900716, AY900717, AY900718, AY900719, AY900720, AY900721, AY900722, AY900723, AY900724, AY900725, AY900726, AY900727, AY900728, AY900729, AY900730, AY900731, AY900732, AY900733, AY900734, AY900735, AY900736, AY900737, AY900738, AY900739, AY900740, AY900741, AY900742, AY900743, AY900744, AY900745, AY900746, AY900747, AY900748, AY900749, AY900750, AY900751, AY900752, AY900753, AY900754, AY900755, AY900756, AY900757, AY900758, AY900759, AY900760, AY900761, AY900762, AY900763, AY900764, AY900765, AY900766, AY900767, AY900768, AY900769, AY900770, AY900771, AY900772, AY900773, AY900774, AY900775, AY900776, AY900777, AY900778, AY900779, AY900780, AY900781, AY900782,

AY900783, AY900784, AY900785, AY900786, AY900787, AY900788, AY900789, AY900790, AY900791, AY900792, AY900793, AY900794, AY900795, AY900796, AY900797, AY900798, AY900799, AY900800, AY900801, AY900802, AY900803, AY900804, AY900805, AY900806, AY900807, AY900808, AY900809, AY900810, AY900811, AY900812, AY900813, AY900814, AY900815, AY900816, AY900817, AY900818, AY900819, AY900820, AY900821, AY900822, AY900823, AY900824, AY900825, AY900826, AY900827, AY900828, AY900829, AY900830, AY900831, AY900832, AY900833, AY900834, AY900835, AY900836, AY900837, AY900838, AY900839, AY900840, AY900841, AY900842, AY900843, AY900844, AY900845, AY900846, AY900847, AY900848, AY900849, AY900850, AY900851, AY900852, AY900853, AY900854, AY900855, AY900856, AY900857, AY900858, AY900859, AY900860, AY900861, AY900862, AY900863, AY900864, AY900865, AY900866, AY900867, AY900868, AY900869, AY900870, AY900871, AY900872, AY900873, AY900874, AY900876, AY900877, AY900879, AY900880, AY900881, AY900882, AY900883, AY900885, AY900887, AY900888, AY900889, AY900890, AY900891, AY900892, AY900893, AY900894, AY900895, AY900896, AY900897, AY900898, AY900899, AY900900, AY900901, AY900902, AY900903, AY900904, AY900905, AY900906, AY900907, AY900908, AY900909, AY900910, AY900911, AY900912, AY900913, AY900914, AY900915, AY900916, AY900917, AY900918, AY900919, AY900920, AY900921, AY900922, AY900923, AY900924, AY900925, AY900926, AY900927, AY900928, AY900929, AY900930, AY900931, AY900932, AY900933, AY900934, AY900935, AY900936, AY900937, AY900938, AY900939, AY900940, AY900941, AY900942, AY900943, AY900944, AY900945, AY900946, AY900947, AY900948, AY900949, AY900950, AY900951, AY900952, AY900953, AY900954, AY900955, AY900956, AY900957, AY900958, AY900959, AY900960, AY900961, AY900962, AY900963, AY900964, AY900965, AY900966, AY900967, AY900968, AY900969, AY900970, AY900971, AY900972, AY900973, AY900974, AY900975, AY900976, AY900977, AY900978, AY900979, AY900980, AY900981, AY900982, AY900983, AY900984, AY900985, AY900986, AY900987, AY900988, AY900989, AY900990, AY900991, AY900992, AY900993, AY900994, AY900995, AY900996, AY900997, AY900998, AY900999, AY901000, AY901001, AY901002, AY901003, AY901004, AY901005, AY901006, AY901007, AY901008, AY901009, AY901010, AY901011, AY901012, AY901013, AY901014, AY901015, AY901016, AY901017, AY901018, AY901019, AY901020, AY901021, AY901022, AY901023, AY901024, AY901025, AY901026, AY901027, AY901028, AY901029, AY901030, AY901031, AY901032, AY901033, AY901034, AY901035, AY901036, AY901037, AY901038, AY901039, AY901040, AY901041, AY901042, AY901043, AY901044, AY901045, AY901046, AY901047, AY901048, AY901049, AY901050, AY901051, AY901052, AY901053, AY901054, AY901055, AY901056, AY901057, AY901058, AY901059, AY901060, AY901061, AY901062, AY901063, AY901064, AY901065, AY901066, AY901067, AY901068, AY901069, AY901070, AY901071, AY901072, AY901073, AY901074, AY901075, AY901076, AY901077, AY901078, AY901079, AY901080, AY901081, AY901082, AY901083, AY901084, AY901085, AY901086, AY901087, AY901088, AY901089, AY901090, AY901091, AY901092, AY901093, AY901094, AY901095, AY901096, AY901099, AY901100, AY901101, AY901102, AY901103, AY901104, AY901105, AY901106, AY901107, AY901108, AY901109, AY901181,

AY901182, AY901183, AY901184, AY901203, AY901204, AY901205, AY901206, AY901207, AY901208, AY901209, AY901210, AY901211, AY901212, AY901213, AY901214, AY901215, AY901216, AY901217, AY901218, AY901219, AY901220, AY901221, AY901222, AY901223, AY901224, AY901225, AY901226, AY901227, AY901228, AY901229, AY901230, AY901231, AY901232, AY901233, AY901234, AY901235, AY901236, AY901237, AY901238, AY901239, AY901240, AY901241, AY901242, AY901243, AY901244, AY901245, AY901246, AY901247, AY901248, AY901249, AY901250, AY901251, AY901252, AY901253, AY901254, AY901255, AY901256, AY901257, AY901258, AY901259, AY901260, AY901261, AY901262, AY901263, AY901264, AY901265, AY901266, AY901267, AY901268, AY901269, AY901270, AY901271, AY901272, AY901273, AY901274, AY901275, AY901276, AY901277, AY901278, AY901279, AY901280, AY901281, AY901282, AY901283, AY901284, AY901285, AY901286, AY901287, AY901288, AY901289, AY901290, AY901291, AY901292, AY901293, AY901294, AY901295, AY901296, AY901297, AY901298, AY901299, AY901300, AY901301, AY901302, AY901303, AY901304, AY901305, AY901306, AY901307, AY901308, AY901309, AY901310, AY901311, AY901312, AY901313, AY901314, AY901318, AY901319, AY901320, AY901321, AY901322, AY901323, AY901324, AY901325, AY901326, AY901327, AY901328, AY901329, AY901330, AY901331, AY901332, AY901333, AY901334, AY901335, AY901336, AY901337, AY901338, AY901339, AY901340, AY901341, AY901342, AY901343, AY901344, AY901345, AY901346, AY901347, AY901348, AY901349, AY901350, AY901351, AY901352, AY901353, AY901354, AY901355, AY901356, AY901357, AY901358, AY901359, AY901360, AY901361, AY901362, AY901363, AY901364, AY901365, AY901366, AY901367, AY901368, AY901369, AY901370, AY901371, AY901372, AY901373, AY901374, AY901375, AY901376, AY901377, AY901378, AY901379, AY901380, AY901381, AY901382, AY901383, AY901384, AY901385, AY901386, AY901387, AY901388, AY901389, AY901390, AY901391, AY901392, AY901393, AY901394, AY901395, AY901396, AY901397, AY901398, AY901399, AY901400, AY901401, AY901402, AY901403, AY901404, AY901405, AY901406, AY901407, AY901408, AY901409, AY901410, AY901411, AY901412, AY901413, AY901414, AY901415, AY901416, AY901417, AY901418, AY901419, AY901420, AY901421, AY901422, AY901423, AY901424, AY901425, AY901426, AY901427, AY901429, AY901430, AY901432, AY901433, AY901434, AY901435, AY901436, AY901439, AY901440, AY901441, AY901442, AY901443, AY901444, AY901445, AY901446, AY901447, AY901448, AY901449, AY901450, AY901451, AY901452, AY901453, AY901454, AY901455, AY901456, AY901457, AY901458, AY901459, AY901460, AY901461, AY901462, AY901463, AY901464, AY901465, AY901466, AY901467, AY901468, AY901469, AY901470, AY901471, AY901472, AY901473, AY901474, AY901475, AY901476, AY901477, AY901478, AY901479, AY901480, AY901481, AY901482, AY901483, AY901484, AY901485, AY901486, AY901487, AY901488, AY901489, AY901490, AY901491, AY901492, AY901493, AY901494, AY901495, AY901496, AY901497, AY901498, AY901499, AY901500, AY901501, AY901502, AY901503, AY901504, AY901505, AY901506, AY901507, AY901508, AY901509, AY901510, AY901511, AY901512, AY901513, AY901514, AY901515, AY901516, AY901517, AY901518, AY901519, AY901520, AY901521, AY901522, AY901523, AY901524, AY901525, AY901526, AY901527, AY901528,

AY901529, AY901530, AY901531, AY901532, AY901533, AY901534, AY901535,
AY901536, AY901537, AY901538, AY901539, AY901540, AY901541, AY901542,
AY901543, AY901544, AY901545, AY901546, AY901547, AY901548, AY901549,
AY901550, AY901551, AY901552, AY901553, AY901554, AY901555, AY901556,
AY901557, AY901558, AY901559, AY901560, AY901561, AY901562, AY901563,
AY901564, AY901565, AY901566, AY901567, AY901568, AY901569, AY901570,
AY901571, AY901572, AY901573, AY901574, AY901575, AY901576, AY901577,
AY901578, AY901579, AY901580, AY901581, AY901582, AY901583, AY901584,
AY901585, AY901586, AY901587, AY901588, AY901589, AY901590, AY901591,
AY901592, AY901593, AY901594, AY901595, AY901596, AY901597, AY901598,
AY901599, AY901600, AY901601, AY901602, AY901603, AY901604, AY901605,
AY901606, AY901607, AY901608, AY901609, AY901610, AY901611, AY901612,
AY901613, AY901614, AY901615, AY901616, AY901617, AY901618, AY901619,
AY901620, AY901621, AY901622, AY901623, AY901624, AY901625, AY901626,
AY901627, AY901628, AY901629, AY901630, AY901631, AY901632, AY901633,
AY901634, AY901635, AY901636, AY901637, AY901638, AY901639, AY901640,
AY901641, AY901642, AY901643, AY901644, AY901645, AY901646, AY901647,
AY901648, AY901649, AY901650, AY901651, AY901652, AY901653, AY901654,
AY901655, AY901656, AY901657, AY901658, AY901659, AY901660, AY901661,
AY901662, AY901663, AY901664, AY901665, AY901666, AY901667, AY901668,
AY901669, AY901670, AY901671, AY901672, AY901673, AY901674, AY901675,
AY901676, AY901677, AY901678, AY901679, AY901680, AY901681, AY901682,
AY901683, AY901684, AY901685, AY901686, AY901687, AY901688, AY901689,
AY901690, AY901691, AY901692, AY901693, AY901694, AY901695, AY901696,
AY901697, AY901698, AY901699, AY901700, AY901701, AY901702, AY901703,
AY901704, AY901705, AY901709, AY901710, AY901711, AY901712, AY901713,
AY901714, AY901715, AY901716, AY901717, AY901718, AY901719, AY901720,
AY901721, AY901722, AY901723, AY901724, AY901725, AY901726, AY901727,
AY901728, AY901729, AY901730, AY901731, AY901732, AY901733, AY901734,
AY901735, AY901736, AY901737, AY901738, AY901739, AY901740, AY901741,
AY901742, AY901743, AY901744, AY901745, AY901746, AY901747, AY901748,
AY901749, AY901750, AY901751, AY901752, AY901753, AY901754, AY901755,
AY901756, AY901757, AY901758, AY901759, AY901760, AY901761, AY901762,
AY901763, AY901764, AY901765, AY901766, AY901767, AY901768, AY901769,
AY901770, AY901771, AY901772, AY901773, AY901774, AY901775, AY901776,
AY901777, AY901778, AY901779, AY901780, AY901781, AY901782, AY901783,
AY901784, AY901785, AY901786, AY901808, AY901809, AY901810, AY901811,
AY901812, AY901813, AY901814, AY901815, AY901816, AY901817, AY901818,
AY901819, AY901820, AY901821, AY901822, AY901823, AY901824, AY901825,
AY901826, AY901827, AY901828, AY901829, AY901830, AY901831, AY901832,
AY901833, AY901834, AY901835, AY901836, AY901837, AY901838, AY901839,
AY901840, AY901841, AY901842, AY901843, AY901844, AY901845, AY901846,
AY901847, AY901848, AY901849, AY901850, AY901851, AY901852, AY901853,
AY901854, AY901855, AY901856, AY901857, AY901858, AY901859, AY901860,
AY901861, AY901862, AY901863, AY901864, AY901865, AY901866, AY901867,
AY901868, AY901869, AY901870, AY901871, AY901872, AY901873, AY901874,

AY901875, AY901876, AY901877, AY901878, AY901879, AY901880, AY901881, AY901882, AY901883, AY901884, AY901885, AY901886, AY901887, AY901888, AY901889, AY901890, AY901891, AY901892, AY901893, AY901894, AY901895, AY901896, AY901897, AY901898, AY901899, AY901900, AY901901, AY901902, AY901903, AY901904, AY901905, AY901906, AY901907, AY901908, AY901909, AY901910, AY901911, AY901912, AY901913, AY901914, AY901915, AY901916, AY901917, AY901918, AY901919, AY901920, AY901921, AY901922, AY901923, AY901924, AY901925, AY901926, AY901927, AY901928, AY901929, AY901930, AY901931, AY901932, AY901933, AY901934, AY901935, AY901936, AY901937, AY901938, AY901939, AY901940, AY901941, AY901942, AY901943, AY901944, AY901945, AY901946, AY901947, AY901948, AY901949, AY901950, AY901951, AY901952, AY901953, AY901954, AY901955, AY901956, AY901957, AY901958, AY901959, AY901960, AY901961, AY901962, HIM24923, K03454, L08456, L08457, L39106, M22639, M27323, M62320, U12741, U12743, U12744, U19416, U19432, U21121, U31242, U31243, U31244, U31245, U31246, U46016, U51188, U51189, U51190, U52953, U54771, U83603, U83604, U83605, U83606, U83607, U83608, U83609, U83610, U83611, U83612, U83613, U83614, U86777, U86780, U88822, U88824, U88826, U92049, Y16138, Y16140, Y16141, Y16142, Y16143, Y16144, Y16145, Y16146, Y16147, Y16148, Y16149, Y16150, Y16151
